# GPER1 Activation Exerts Anti-Tumor Activity in Multiple Myeloma

**DOI:** 10.3390/cells12182226

**Published:** 2023-09-07

**Authors:** Maria Eugenia Gallo Cantafio, Roberta Torcasio, Francesca Scionti, Maria Mesuraca, Domenica Ronchetti, Mariaelena Pistoni, Dina Bellizzi, Giuseppe Passarino, Eugenio Morelli, Antonino Neri, Giuseppe Viglietto, Nicola Amodio

**Affiliations:** 1 Department of Experimental and Clinical Medicine, Magna Graecia University, 88100 Catanzaro, Italy; mariaeugenia.gallocantafio@unicz.it (M.E.G.C.); roberta.torcasio@studenti.unicz.it (R.T.); mes@unicz.it (M.M.); viglietto@unicz.it (G.V.); 2 Laboratory of Cellular and Molecular Cardiovascular Pathophysiology, Department of Biology, Ecology and Earth Sciences (DiBEST), University of Calabria, Arcavacata di Rende, 87036 Cosenza, Italy; 3 Department of Medical and Surgical Science, University Magna Graecia, 88100 Catanzaro, Italy; scionti@unicz.it; 4 Department of Oncology and Hemato-Oncology, University of Milan, 20141 Milan, Italy; domenica.ronchetti@unimi.it; 5 Laboratory of Translational Research, Azienda USL-IRCCS di Reggio Emilia, 42123 Reggio Emilia, Italy; mariaelena.pistoni@ausl.re.it; 6 Department of Biology, Ecology and Earth Sciences, University of Calabria, 87036 Rende, Italy; dina.bellizzi@unical.it (D.B.); giuseppe.passarino@unical.it (G.P.); 7 Jerome Lipper Multiple Myeloma Center, Department of Medical Oncology, Dana-Farber Cancer Institute, Boston, MA 02215, USA; eugenio_morelli@dfci.harvard.edu; 8 Scientific Directorate, Azienda USL-IRCCS di Reggio Emilia, 42123 Reggio Emilia, Italy; antonino.neri@ausl.re.it

**Keywords:** GPER, multiple myeloma, G-1, plasma cell dyscrasias

## Abstract

G protein-coupled estrogen receptor 1 (GPER1) activation is emerging as a promising therapeutic strategy against several cancer types. While GPER targeting has been widely studied in the context of solid tumors, its effect on hematological malignancies remains to be fully understood. Here, we show that GPER1 mRNA is down-regulated in plasma cells from overt multiple myeloma (MM) and plasma cell leukemia patients as compared to normal donors or pre-malignant conditions (monoclonal gammopathy of undetermined significance and smoldering MM); moreover, lower GPER1 expression associates with worse overall survival of MM patients. Using the clinically applicable GPER1-selective agonist G-1, we demonstrate that the pharmacological activation of GPER1 triggered in vitro anti-MM activity through apoptosis induction, also overcoming the protective effects exerted by bone marrow stromal cells. Noteworthy, G-1 treatment reduced in vivo MM growth in two distinct xenograft models, even bearing bortezomib-resistant MM cells. Mechanistically, G-1 upregulated the miR-29b oncosuppressive network, blunting an established miR-29b-Sp1 feedback loop operative in MM cells. Overall, this study highlights the druggability of GPER1 in MM, providing the first preclinical framework for further development of GPER1 agonists to treat this malignancy.

## 1. Introduction

Multiple myeloma (MM) is a B-cell malignancy of clonal plasma cells (PCs) accumulating in the bone marrow (BM), underlined by an intricate genomic, epigenomic, and immune landscape [1,2,3]. Despite significant advancements in the pathobiology of the disease, which have increased the extent and frequency of response with remarkable outcome improvement, most MM patients progress to a drug-resistant phase culminating in death [4,5]. On this basis, the identification of new anti-MM druggable vulnerabilities remains a major goal in this field. 

The G protein-coupled estrogen receptor 1 (GPER1) is a 7-transmembrane domain membrane estrogen receptor (ER), located mainly at the plasma membrane when N-terminal glycosylated, and correctly folded in the endoplasmic reticulum membrane [6]. Ligand stimulation of GPER1 leads to a conformational change of the receptor, allowing a direct interaction between GPER1 and heterotrimeric G_s proteins, followed by activation of various non-genomic signaling pathways [7,8,9].

Studies carried out with GPER1-deficient mice, or exploiting GPER1-selective agonists, have shown that GPER1-signaling plays an important function in various physio-pathological conditions, including cancer [10]. In this regard, GPER1 has been implicated in non-genomic estrogenic signaling, including calcium mobilization and generation of cyclic AMP, and stimulation of GPER1 can modulate matrix metalloproteinases, epidermal growth factor receptor (EGFR), ERK, and PI3K pathways [10].

GPER1 has been found expressed in cancers, where its role has been extensively investigated especially in solid tumors, in which a dualistic tumor-promoting or -suppressive function emerges, depending on the tumor type. In certain female neoplasms, higher GPER1 expression is associated with inferior prognosis and contributes to tumor development [11,12,13,14]; conversely, GPER1-dependent anti-tumor effects have been reported in other tumor types, as demonstrated by the tumor-inhibiting effects elicited by the selective small-molecule agonist G-1, or by its enantiomer LNS8801, in glioblastoma [15], Leydig tumor cells [16], uveal melanoma [17], and many others [18].

In the context of hematological malignancies, the role and the therapeutic potential of GPER1-targeting have been less investigated. In acute myeloid leukemia (AML), GPER1 expression is significantly down-regulated in tumor blasts, and its pharmacological activation via G-1 agonist impairs in vitro and in vivo leukemogenesis, improving CD8^+^ T cell functions and enhancing venetoclax activity [19]; similarly, the cytotoxic activity of G-1 was reported against T-cell lymphoblastic leukemia [20]. Preclinical studies on mantle cell lymphoma (MCL) and Waldenström macroglobulinemia (WM) also indicate that G-1-mediated selective GPER1 activation exerts anti-tumor activity through ROS production, NF-κB inhibition [21], or p53 upregulation [22].

In this study, for the first time, we investigated the expression pattern of GPER1 in plasma cell dyscrasias, and the functional sequelae underpinning its selective activation using in vitro and in vivo models of MM, a still incurable plasma cell malignancy [23]. Overall, our data demonstrate that G-1 exerts potent anti-MM activity, even capable of overcoming the protective BM *milieu*, modulating an established miRNA-transcription factor loop, i.e., the miR-29b-Sp1, operative in MM. 

## 2. Materials and Methods

### 2.1. Gene Expression Profiling Analysis

The gene expression profiling of *GPER1* was evaluated in four publicly available datasets of purified plasma cells samples including 24 normal donors (N), 34 monoclonal gammopathy of undetermined significance (MGUS), 24 smoldering MM (SMM), 172 MM, and 9 plasma cell leukemia (PCL) patients, together with 23 human myeloma cell lines (HMCLs). All samples were profiled on GeneChip^®^ Human Genome U133A Arrays (Affymetrix, Santa Clara, CA, USA) and deposited at the National Center for Biotechnology Information’s Gene Expression Omnibus (GEO) (https://www.ncbi.nlm.nih.gov/geo/, accessed on 14 July 2023) with the GEO Series accession numbers GSE13591 [24], GSE6477 [25], GSE6691 [26], and GSE6205 [27]. CEL files were downloaded and expression data were generated using the Robust Multi-Array average (RMA) procedure as previously described [28]. Overall survival (OS) analysis was performed on the GSE9782 dataset, including a cohort of 264 relapsed untreated MM patients from Mulligan et al. [29], available on the public gene expression website GenomicScape (http://www.genomicscape.com/, accessed on 14 July 2023). 

### 2.2. Cell Lines and Primary Samples

NCI-H929, RPMI-8226, U266, SKMM1, MM1s, MM1R, and JJN3 MM cell lines were purchased from DSMZ; KMS11 and OPM2 cell lines were obtained by the Japanese Collection of Research Bioresources (National Institute of Health Sciences, Kawasaki, Kanagawa, Japan). AMO-wt, AMO-CFZ, and AMO-BZB cells were provided by Dr. C. Driessen (University of Tubingen, Baden-Württemberg, Germany). INA-6 cell line was previously obtained by Dr. Renate Burger (University of Erlangen-Nurnberg, Erlangen, Germany). All these cell lines were immediately frozen and used from the original stock within 6 months. HMCLs were cultured in RPMI-1640 medium (Corning), supplemented with 10% heat-inactivated FBS (Gibco, Life Technologies, Carlsbad, CA, USA), 100 U/mL penicillin, 100 mg/mL streptomycin (Gibco, Life Technologies, Carlsbad, CA, USA), and incubated at 37 °C in a 5% CO_2_ atmosphere. The IL-6-dependent MM cell line INA-6 was cultured in the presence of recombinant human IL-6 (R&D Systems, Minneapolis, MN, USA). Bone marrow mononuclear cells and primary CD138^+^ cells from bone marrow aspirates of newly diagnosed MM (NDMM) patients were isolated by using the Ficoll-Hypaque density gradient separation method (Lonza Group, Basel, Switzerland). Following informed consent of all patients and Institutional Ethical Committee approval (Institutional Approval: n. 266/2021), primary CD138^+^ cells were isolated from MM patient-derived bone marrow aspirate by antibody-mediated positive selection using anti-CD138 microbeads kit (Miltenyi Biotec, Gladbach, Germany). The purity of immunoselected cells was assessed by FACS analysis using PE-conjugated CD138 antibody (BD Bioscience, San Jose, CA, USA). The residual CD138^-^ bone marrow mononuclear cells were cultured in RPMI-1640 supplemented with 20% FBS for up to 6 weeks to generate bone marrow stromal cells (BMSCs). Peripheral blood mononuclear cells (PBMCs) were isolated from healthy donors’ peripheral blood by using the Ficoll-Hypaque density gradient separation method. Cells were periodically tested for mycoplasma contamination. 

### 2.3. Drugs

G-1 and G-15 were purchased from Tocris, dissolved in dimethyl sulfoxide (DMSO) at 10 mg/mL, and stored at −20 °C. Bortezomib was purchased from Selleck Chemicals LLC (Munich, Germany).

### 2.4. Cell Viability and Proliferation Assays 

Cell viability was evaluated by using the Cell Titer Glo (CTG) assay (Promega, Madison, WI, USA), as reported [30]. MM cells were seeded in 24-well plates, treated with different concentrations of G-1 alone or in combination with G-15 or Z-VAD, and collected at different time points. Luminescence was measured in a white plate by using a GloMax-multi detection system (Promega, Madison, WI, USA). 

Cell proliferation was evaluated by BrdU assay. MM cells were seeded in 96-well plates and treated with different G-1 doses; BrdU incorporation was evaluated at 48 h, by measuring luminescence using a Victor 4 plate reader (Perkin Elmer, Waltham, MA, USA). Each sample was run at least in triplicate. 

### 2.5. Apoptosis Assays

Apoptosis and cell viability were investigated by using Annexin V/7-Amminoactinomycin (7-AAD) flow cytometry assay, as previously reported [31]. MM cells were stained in a 5 mL polystyrene tube according to PE/Annexin V Apoptosis detection kit (Thermo Fisher Scientific, Waltham, MA, USA) protocol. Briefly, MM cells were harvested after treatments, washed in PBS 1×, then exposed to Annexin V-PE and 7-AAD probes, and incubated at room temperature for 15 min. Sample analysis was performed by using FACS Fortessa X-20 (BD Bioscience, San Jose, CA, USA). Results were analyzed by using FlowJo software version 10 and reported as histogram bars as the percentage of total apoptotic cells.

The caspase activity in MM-treated cells was evaluated by using the Caspase-Glo 3/7 assay kit (Promega, Madison, WI, USA), according to the manufacturer’s instructions. The assay provides a proluminescent caspase-3/7 substrate in a buffer system optimized for the measurement of caspase activity in cell lysates. Briefly, MM cells were seeded in a white 96-well plate in triplicate. After 48 h from treatment, the assay reagent was added to the well and incubated at room temperature for 60 min; luminescence was measured using the GloMax-multi detection system (Promega, Madison, WI, USA). 

The assays were performed in triplicate and represented as means ± SD for each condition.

### 2.6. Lentivirus Production, Transduction and Transfection of MM Cells

To obtain MM cells stably expressing luciferase transgene for in vivo study, AMO-BZB cells were infected with pLenti-III-PGK-Luc Control Vector according to the manufacturer’s instructions (Applied Biological Materials, Richmond, Canada); two days after transduction, luciferase positive cells were selected using 0.5 µg/mL puromycin for 3 weeks.

### 2.7. Reverse Transcription and Quantitative Real-Time Amplification (q-RT-PCR)

RNA extraction, reverse transcription (RT), and quantitative real-time amplification (q-RT-PCR) were performed as previously described [32]. Briefly, total RNA was isolated from MM cells by using TRIzol Reagent (Thermo Fisher Scientific, Waltham, MA, USA), according to the manufacturer’s protocol. The integrity and purity of total RNA isolated were measured by using Nanodrop (Celbio Nanodrop Spectrophotometer nd-2000, Thermo Fisher Scientific, Waltham, MA, USA). 

The single-tube TaqMan miRNA assay (Applied Biosystems, Thermo Fisher Scientific, Waltham, MA, USA) was used to detect and quantify mature miR-29b (Assay ID: 000413, Applied Biosystems, Life Technologies) according to the manufacturer’s instructions, by the use of QuantStudio 12K Flex reader (Thermo Fisher Scientific, Waltham, MA, USA). miR-29b expression was normalized on RNU44 (Applied Biosystems, assay ID: 001094). 

### 2.8. Western Blotting (WB)

Total proteins were extracted using NP40 Cell Lysis Buffer supplemented with Halt Protease Inhibitor Single-Use Cocktail (Thermo Fisher Scientific, Waltham, MA, USA). For WB analysis, 40 µg of whole cell lysate of each sample were separated by electrophoresis on NuPAGE gels (precast, 4–12%; Invitrogen—Thermo Fisher Scientific, Waltham, MA, USA) and electro-transferred to nitrocellulose membrane using the Trans-Blot Turbo Transfer System (Bio-Rad Laboratories, Hercules, CA, USA), as reported [32]. After protein transfer, the membranes were immunoblotted with each of the following antibodies: Caspase 3 (#9665P), Caspase 7 (#12827P), PARP (#9532), Sp1 (#9389S), MCL-1 (#5453S), CDK6 (#13331), GAPDH (#5174), and α-tubulin (#2125S), all purchased from Cell Signaling Technology; or GPER1 (ab154069) from Abcam (Cambridge, UK).

### 2.9. Chromatin Immunoprecipitation (ChIP)

MM cells (1.5 × 10^7^) were crosslinked in 1% formaldehyde, lysed, and sheared by sonication for 10 cycles (30 s each) on a cold block with intervals of 90 s of cooling by using the Bioruptor Plus (Diagenode, Denville, NJ, USA). Chromatin was divided into equal amounts of immunoprecipitation with Sp1 antibody (Abcam, cat#24543), or rabbit IgG as a negative control (Santa Cruz Biotechnology, Dallas, TX, USA). Chromatin extracts were incubated on a rotator with 20 µL of ChIP Grade Protein A/G Plus Agarose for 3 h at 4 °C. Bound agarose beads were then harvested by centrifugation (12,000 rpm, 15 s) and washed. The precipitated protein-DNA complexes were separated from beads and incubated twice at 65 °C for 1.5 h with NaCl and Proteinase K to revert cross-links. Purified DNA was subjected to qPCR using GoTaq qPCR Master Mix (Promega, Madison, WI, USA). Primer sequences for qPCR were the following: *GPER* promoter Sp1 binding site 1- Fw AGTAGGTCTTGGAGACGGAAGT and *GPER* promoter Sp1 binding site 1- Rev: CAGCATAGAGTCAGCGGATCG.

### 2.10. In Vivo Studies

CB-17 severe combined immunodeficient (SCID) mice, which were 5–6 weeks old, were purchased from Envigo Company and housed and monitored in the animal facility at Dana-Farber Cancer Institute (DFCI). All experiments were performed after approval by the Animal Ethics Committee of the DFCI and performed using institutional guidelines. In the first experimental model, mice were subcutaneously inoculated with 5 × 10^6^ MM1S cells in 100 µL of RPMI-1640. When tumors became palpable (100–200 mm^3^; ≈3 weeks after injection), mice were divided into two groups (n = 5/group), and intraperitoneally treated with G-1 (1 mg/kg body weight) daily for 4 days a week, for 3 weeks. The control group received vehicles alone by using the same schedule as the G-1 group. Tumor size was measured twice a week by using an electronic caliper and calculated with the following formula: *V* = (*a*^2^ × *b*)/2, where *a* is the width and *b* is the length of the tumor.

In the second model, animals were subcutaneously inoculated with 5 × 10^6^ luciferase-engineered AMO-BZB MM cells. When tumors became palpable, mice were randomized into 3 groups (n = 5/group) to receive intraperitoneal treatments with either vehicle alone (NaCl 0.9% solution) or G-1 (1 mg/kg body weight) daily for 4 days a week, for 3 weeks; bortezomib (1 mg/kg body weight) was given on days 1, 4, 8, and 11. Tumor volume was measured by IVIS Lumina II Imaging System (Caliper Life Sciences, Hopkinton, MA, USA).

Animals were euthanized when tumors achieved the size of 2 mm^3^ or in the presence of events that compromised the animals’ quality of life. 

### 2.11. Statistical Analysis 

Each experiment was performed at least in triplicate and values were reported as mean ± SD. Data were analyzed using Student’s *t*-tests for the comparisons between groups, whereas the statistical significance of differences among multiple groups was calculated by GraphPad software version 9.0 (GraphPad Software, La Jolla, CA, USA). Graphs were obtained using GraphPad Prism version 9.0. Survival analysis was performed using a log-rank test. *p*-value < 0.05 was considered significant. 

## 3. Results

### 3.1. GPER mRNA Is Down-Regulated in MM and Associates with Worse Overall Survival

**Figure 1:**
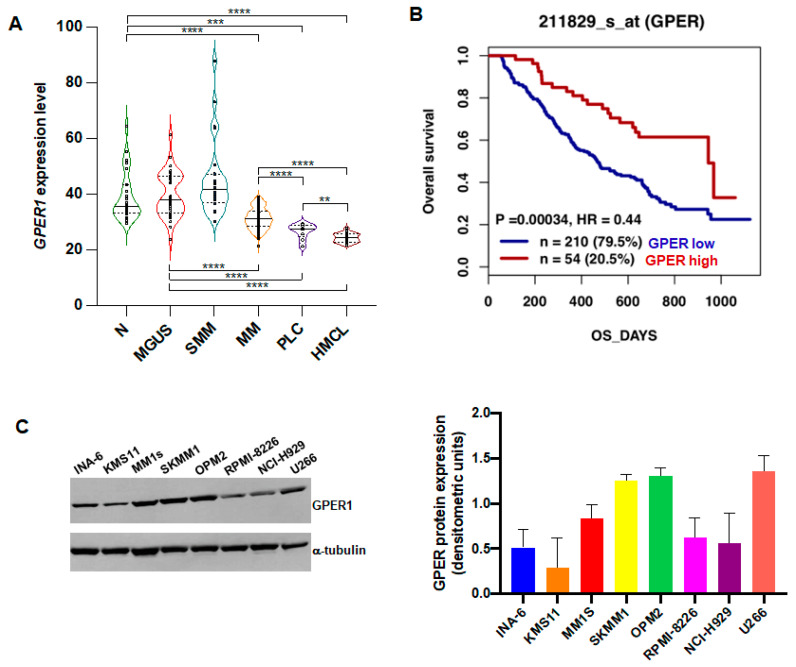
Low GPER1 expression is observed in MM and PCL and associates with poor overall survival. (**A**) Violin plot showing expression levels of GPER1 mRNA in normal samples (n = 24), MGUS (n = 34), SMM (n = 24), MM (n = 172), PCL (n = 9), and HMCLs (n = 23) from GSE6477, GSE6691, GSE13591, and GSE6205 datasets. Each dot represents a single sample. Expression levels were compared using an unpaired Student’s *t*-test. ** *p* ≤ 0.01; *** *p* ≤ 0.001; **** *p* ≤ 0.0001. N = normal donors; MGUS = monoclonal gammopathy of undetermined significance; MM = multiple myeloma; SMM = smoldering MM; PCL = plasma cell leukemia; HMCLs = human MM cell lines. (**B**) Kaplan-Meyer analysis of the overall survival of 264 MM patients in the Mulligan dataset (GSE9782) with low (blue) and high (red) GPER1 expression levels. Survival analysis was performed using the log-rank test. (**C**) WB analysis of GPER1 protein expression in MM cell lines. Densitometric analysis of protein expression in three different experiments is reported on the right; α-tubulin was used as a loading control.

Firstly, we interrogated the transcriptome profiles of four GEO datasets to analyze the expression level of GPER1 in different subgroups of plasma cell dyscrasias patients and in normal controls. We found a significantly reduced expression of GPER1 mRNA in symptomatic myeloma (MM) and in the more aggressive extra-medullary stages of plasma cell dyscrasias (PCL) with respect to normal PCs, or to PCs from MM pre-malignant conditions (MGUS and SMM), with the lowest expression observed in the HMCLs group (Figure 1A). These results prompted us to explore the prognostic role of GPER1 in MM. The analysis of GSE9782 from the Mulligan dataset revealed a significant association of GPER1 with clinical outcome: indeed, MM patients with lower GPER1 expression had shorter OS than those with higher expression (Figure 1B). These findings suggest a potential role of GPER1 expression in predicting MM progression as well as patient survival. Finally, we explored, by Western blot, GPER1 protein expression in a panel of MM cell lines. Our results showed that GPER1 was detectable in all the cell lines tested (Figure 1C), prompting us to investigate its functional role using selective agonists or antagonists.

### 3.2. In Vitro Anti-Tumor Activity of the GPER1 Agonist G-1 in MM

**Figure 2:**
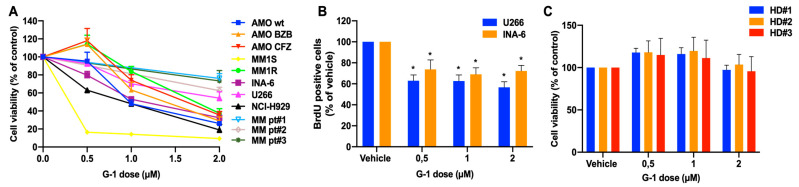
G-1 triggers anti-MM activity in vitro. (**A**) Cell viability was assessed by CTG assay in MM cell lines as well as in three different NDMM patient-derived plasma cells (MM pt#1, MM pt#2, and MM pt#3), 48 h after G-1 treatment. (**B**) BrdU incorporation assay was performed in U266 and INA-6 MM cell lines after 48 h of G-1 exposure. Data represent the average of at least three independent experiments. * *p* < 0.05. (**C**) Cell viability was assessed by CTG assay on PBMCs derived from three different healthy donors (HD), 48 h after G-1 treatment.

To assess the biological impact of GPER1 activation on MM cells, we exploited the selective small molecule G-1, which has been previously demonstrated as a potent agonist of GPER1 in different tumor types [21,22,33]. Exposure to low micromolar concentrations of G-1 strongly impaired the viability of several MM cell lines, representative of different MM cytogenetics abnormalities, as well as primary PCs from three newly diagnosed MM patients, as assessed by CTG assay (Figure 2A; IC50 values are reported in Supplementary Table S1). The BrdU incorporation assay produced similar results, showing the reduction of INA-6 and U266 cell proliferation 48 h after G-1 treatment (Figure 2B). The inhibitory effects of G-1 on MM cells were partially abrogated after GPER1 inhibition by G-15, a selective GPER1 antagonist (Supplementary Figure S1). Conversely, no cytotoxic effects were observed on PBMCs isolated from three different healthy donors (Figure 2C). 

### 3.3. G-1 Is Pro-Apoptotic in MM

**Figure 3:**
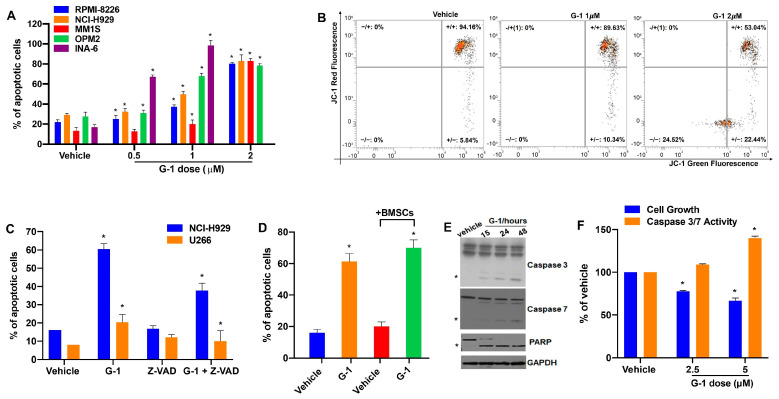
G-1 triggers apoptosis of MM cells. (**A**) FACS analysis of Annexin V/7-AAD stained RPMI-8226, NCI-H929, MM1S, OPM2, and INA-6 MM cell lines after 48 h of G-1 treatment. Histogram bars are representative of the percentage of apoptotic cells in at least three independent experiments. * *p* < 0.05. (**B**) FACS detection of JC-1 fluorescence of NCI-H929 cells, 8 h after treatment with 2 µM G-1 or vehicle. Dot plots are representative of an independent biological replicate (n = 3). (**C**) Histogram bars represent the percentage of apoptotic cells assessed by FACS analysis of Annexin V/7-AAD-labeled U266 and NCI-H929 cells, 48 h after 2 µM G-1 treatment, alone or in combination with 20 µM Z-VAD. * *p* < 0.05. (**D**) FACS analysis of Annexin V-positive NCI-H929 cells, alone or co-cultured with BMSCs, 48 h after 2 µM G-1 treatment. (**E**) WB analysis of Caspase-3/7 and PARP in NCI-H929 cells treated with 2 µM G-1; * indicates the cleaved active form; GAPDH was used as a loading control. (**F**) Cell viability and Caspase 3/7 activities were assessed by CTG assay and Caspase 3/7 Glo assay, respectively, in primary PCs derived from a PCL patient ex-vivo exposed to different concentrations of G-1 for 48 h. * *p* < 0.05.

We next investigated the type of cell death triggered by G-1 in MM. Based on previous studies demonstrating that G-1 is pro-apoptotic in many tumor types [21,22,34,35], we investigated possible apoptotic effects elicited by G-1 in MM cell models. As hypothesized, G-1 induced a dose-dependent increase of apoptotic cells, as assessed by flow cytometry analysis of Annexin V-positive MM cell lines (Figure 3A and Supplementary Figure S2). G-1 pro-apoptotic effects were also maintained in MM cells co-cultured with BMSCs (Figure 3D), underlying G-1’s capability to overcome their protective role on MM cells. Furthermore, apoptosis induction was confirmed by the increase in JC-1-labeled MM cells after 48 h of G-1 exposure (Figure 3B), which indicates mitochondrial membrane potential depolarization occurring in early apoptosis. On the other hand, co-treatment with the pan-caspase inhibitor Z-VAD-fmk partially abrogated the induction of apoptosis induced by G-1 activation in NCI-H929 and U266 MM cell lines, assessed by FACS analysis of Annexin-V positive cells (Figure 3C). Finally, WB analysis confirmed apoptosis activation through the cleavage of Caspase-3/7 and PARP after G-1 treatment in NCI-H929 cells (Figure 3E). Worthy of note, apoptosis induction, along with a decrease in cell viability, were also observed after ex-vivo treatment of primary PCs isolated from a PCL patient (Figure 3F). 

### 3.4. G-1 Upregulates the miR-29b Network in MM Cells

**Figure 4:**
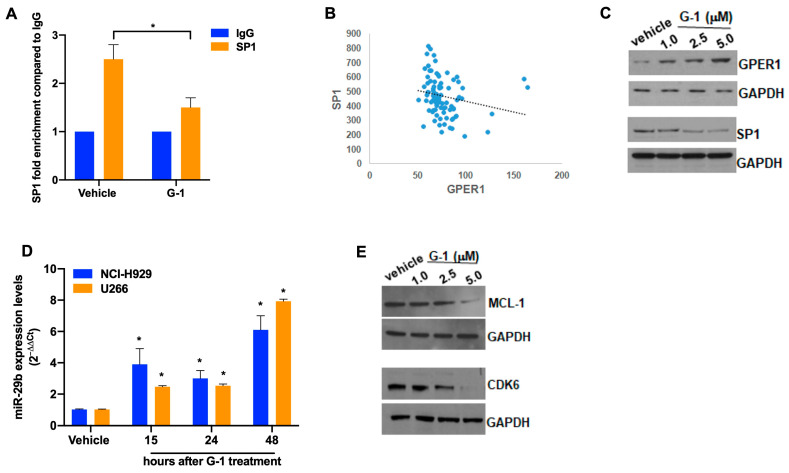
G-1 regulates the miR-29b/Sp1 transcriptional feedback loop. (**A**) qPCR for GPER promoter performed after ChIP with Sp1 antibody, in NCI-H929 cell line treated for 48 h with 2 µM of G-1, or vehicle as control. Data are representative of at least three independent experiments. * *p* < 0.05. (**B**) GSE17498 dataset interrogation showing an inverse correlation between mRNA levels of GPER and Sp1 in MM patients (*p* = 0.009967; R^2^ = 0.0423); linear regression is shown as dotted line. (**C**) WB of GPER1 and SP1 protein levels in NCI-H929 cells, 24 h after G-1 treatment. GAPDH was used as a loading control. (**D**) q-RT-PCR of hsa-miR-29b expression in NCI-H929 cells after 2 µM G-1 exposure; results represent the average of miR-29b expression levels after normalization using RNU44 housekeeping and ΔΔCt calculations; * *p* < 0.05. (**E**) WB of MCL1 and CDK6 protein levels in NCI-H929 cells, 24 h after G-1 treatment. GAPDH was used as a loading control.

We next investigated the molecular network underlying GPER1 activation in MM. Previous studies indicated the presence, in the GPER1 promoter, of binding sites for Sp1 [36], an oncogenic transcription factor highly expressed in MM [37]. To confirm Sp1 binding to GPER1 promoter in MM cells, we carried out a ChIP assay using Sp1 antibodies in the presence of G-1 or a vehicle, which confirmed the binding of Sp1 to GPER promoter, conversely reduced after G-1 treatment (Figure 4A). Lending support to Sp1-GPER cross-talk, the analysis of the GSE17498 dataset revealed an inverse correlation between GPER1 and Sp1 mRNA levels in MM patients (Figure 4B); furthermore, in agreement with these findings, G-1 treatment resulted in GPER upregulation, while reducing Sp1 protein expression, in NCI-H929 cells (Figure 4C). We previously demonstrated that Sp1, an established target of the tumor suppressor miR-29b, negatively impacts miR-29b expression acting in the context of an actionable transcriptional feedback loop in MM [38]. On this evidence, we asked whether the pharmacological activation of GPER1 via G-1 could in turn affect the expression of miR-29b in MM cells. According to our hypothesis, G-1 treatment upregulated miR-29b (Figure 4D), while down-regulating other miR-29b validated targets [38], such as CDK6 and MCL-1 (Figure 4E and Supplementary Figure S3). Overall, these findings suggest that GPER1 and Sp1 are involved in an intricate molecular feedback loop, which is amenable to pharmacological intervention via G-1.

### 3.5. G-1 Exerts Anti-MM Activity In Vivo

**Figure 5:**
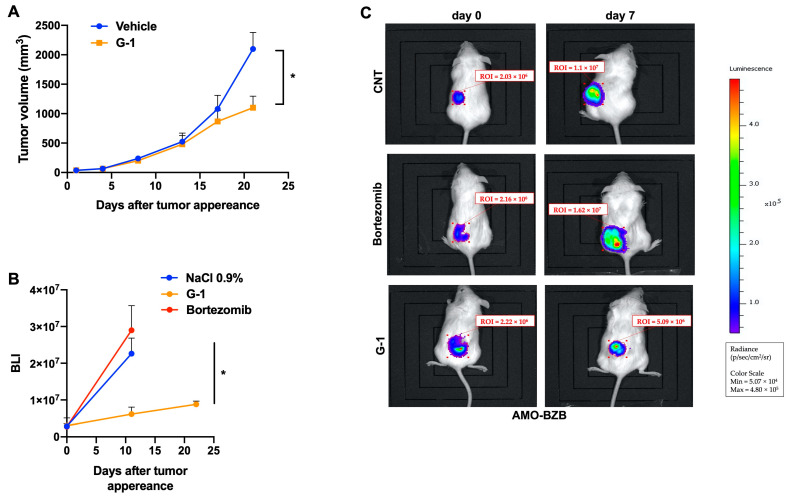
In vivo anti-MM activity of G-1. (**A**) MM1S cells (5 × 10^6^) were subcutaneously implanted in the flank of NOD.SCID mice. When tumors became palpable, mice were randomized and intraperitoneally treated with G-1 (1 mg/kg), 4 days a week for 3 consecutive weeks, or vehicle. Tumor size was measured by using an electron caliper every other day. Averaged tumor volume of each group ±SD is shown (* *p* < 0.05). (**B**) In vivo tumor growth inhibition of luciferase-engineered AMO-BZB subcutaneous xenografts, intraperitoneally treated with G-1 (1 mg/kg), bortezomib (1 mg/kg), or vehicle (NaCl 0.9%). G-1 treatments were performed daily (4 days a week, 3 consecutive weeks), while bortezomib on days 1, 4, 8, and 11. BLI measurements on days 11 and 22 are reported (* *p* < 0.05). (**C**) BLI pictures of a representative G-1-, bortezomib- or vehicle-treated mouse, at day 0 and day 7 after the start of treatments.

The anti-tumor effects of G-1 were finally evaluated in vivo. As a first model, we used NOD.SCID mice bearing a subcutaneous xenograft of MM1S cells. In this model, 5 × 10^6^ MM1S cells were injected into the flank of the mice which, after tumor detection, were randomized into two groups receiving intraperitoneal treatment cycles with G-1 (1 mg/kg), or a vehicle as control. As shown in Figure 5A, G-1 treatment produced a significant reduction of tumor growth as compared to control. In a second in vivo model, we evaluated the anti-tumor activity of G-1 in vivo in MM xenografts resistant to bortezomib, a proteasome inhibitor representing one of the most relevant therapeutic options in MM. NOD.SCID mice were subcutaneously injected with luciferase-engineered bortezomib-resistant AMO-BZB cells (5 × 10^6^); once tumors became palpable, mice were divided into three groups and subjected to intraperitoneal treatment with G-1 (1 mg/kg), bortezomib (1 mg/kg), or vehicle. Bioluminescence was measured, in each group of mice, 7, 11, and 22 days after tumor appearance, demonstrating the efficacy of G-1 in the inhibition of tumor growth (Figure 5B,C). Overall, these data indicate that G-1 could also be effective in the PI refractory settings.

## 4. Discussion

GPER1, also known as GPR30, is a membrane-bound receptor belonging to the G protein-coupled receptor (GPCR) family. Unlike the classical estrogen receptors (ERα and ERβ), GPER1 is not located in the nucleus. It is found on the cell membrane, and its activation triggers various intracellular signaling cascades [39]. 

Unfortunately, despite initial encouraging results from preclinical studies reporting the anti-MM activity of the selective ERα modulator 4-OH-tamoxifen [40], a feasibility clinical trial showed heterogeneous ERα expression in plasmacytosis tumor cells and the lack of activity of tamoxifen [41], thus damping the interest towards ERα as a therapeutic target in MM.

GPER1 expression has been detected in multiple cancer types, where it has been the subject of extensive research due to its implications in tumor development, progression, and response to therapies, with effects that can vary depending on the specific cancer type, its microenvironment, and the interplay with tumor-specific signaling pathways [15,17,42,43,44,45]. As a result, GPER1 is emerging as a novel potential therapeutic target for cancer treatment.

The discovery of GPER1 selective agonists and antagonists has indeed provided insights into GPER1’s pathophysiological role and its involvement in cancer biology [46,47,48]. Depending on the cancer type, GPER1 activation might promote or inhibit carcinogenesis [21]. For example, G-1 reduced liver tumorigenesis by inhibiting inflammation [21], whereas G-1 accelerated tumor growth in mouse models of non-small-cell lung cancer [49]. GPER1 expression has been also implicated in gastric and colon cancers, in which its downregulation is associated with poor prognosis [44,50]. Collectively, GPER1 function has been extensively studied in solid tumors, whereas its role in hematological malignancies is only starting to emerge. Recently, we demonstrated that GPER1 is expressed in WM, and G-1 treatment induced apoptosis of WM cells both in vitro and in vivo, even overcoming the protective bone marrow microenvironment [22].

In the present study, we investigated, for the first time to our knowledge, the GPER1 expression pattern and functional significance in MM. Notably, the expression of GPER1 was significantly down-regulated in PCs from clinically overt diseases, as MM and PCL, with respect to normal PCs or PCs from premalignant conditions (MGUS, SMM), and lower GPER1 expression correlated with inferior overall survival of MM patients. Overall, these data suggest that GPER1 might play a potential tumor suppressor role, as demonstrated in other hematological malignancies such as AML, MCL, and WM, and therefore, its activation can trigger anti-tumor effects. Accordingly, inhibitory effects on MM cell proliferation were elicited by G-1, at low micromolar concentrations, in MM cell lines as well as in primary cells derived from MM and PCL patients. The different G-1 IC50s for HMCLs and primary NDMM cells likely reflect the profound biological heterogeneity of MM cells; future studies are planned to unveil specific biomarkers of G-1 sensitivity to predict MM patients responding to G-1 treatment.

Notably, G-1 also overcame the protective role of BMSCs on MM cells, without affecting the viability of PBMCs from healthy donors, suggesting a favorable therapeutic window. Consistent with these findings, we demonstrated that G-15-mediated antagonism of GPER1 slightly increased MM cell proliferation and partially protected MM cells from G-1-induced cytotoxic effects. Furthermore, our data clearly indicate that GPER1 activation was associated with the induction of apoptosis, as evidenced by alterations in mitochondrial membrane potential and the presence of several apoptotic markers after G-1 treatment. Interestingly, our in vitro results were strengthened by in vivo data, showing that G-1 intraperitoneal treatment could significantly reduce the growth of MM xenografts in two different preclinical models of the disease. 

At the molecular level, our results demonstrate that G-1 treatment affected the previously characterized miR-29b-Sp1 transcriptional feedback loop [38], as suggested by the induction of miR-29b in MM cell lines, and the concomitant down-regulation of Sp1. At the clinical level, this effect was corroborated by the analysis of the GSE17498 dataset, which revealed a negative correlation between GPER and Sp1 mRNA expression in MM patients, underscoring a complex interplay between Sp1 and GPER1, which is currently under further in-depth analysis. Since the miR-29b/Sp1 feedback loop is deregulated in other hematological malignancies, like KIT-driven AML [51,52], it is tempting to speculate that G-1 might trigger anti-tumor effects also in these cancers. Noteworthy, along with Sp1, the reactivation of the oncosuppressive miR-29b was accompanied by the down-regulation of other miR-29b canonical targets, i.e., MCL-1 and CDK6, suggesting a pleiotropic anti-tumor activity of this drug in MM cells. On this evidence, the lack of toxicity of G-1 on healthy PBMCs could be likely ascribed to their low expression of G-1-oncogenic targets as Sp1, CDK6, and MCL-1, conversely upregulated in malignant plasma cells, whose inhibition instead contributes to G-1 anti-MM activity.

In conclusion, the data here presented indicate that GPER1 is a novel druggable target in MM, providing a preclinical rationale for the possible development of GPER1 agonists to tackle this malignancy. G-1 may thus enrich the repertoire of new agents for the treatment of PC dyscrasias.

## Data Availability

The data presented in this study are available upon reasonable request to the corresponding author.

## Supplementary Materials

The following supporting information can be downloaded at: https://www.mdpi.com/article/10.3390/cells12182226/s1, Table S1. IC50 values of G-1 in MM cell lines and primary cells from newly diagnosed MM patients. Cells were treated with different concentrations of G-1 for 48 h, and cell viability assayed using the CTG method. IC50 values were calculated using GraphPad Prism 8 software and reported as mean of three independent experiments ± SD. Figure S1. Cell viability was assessed by CTG assay in NCI-H929 cells, 48 h after 2 µM G-1 treatment, alone or in combination with 0.5 µM G-15. Histogram bars are representative of the percentage of viable cells compared to control. * *p* < 0.05. Figure S2. FACS flow analysis of Annexin V/7-AAD stained RPMI-8226, NCI-H929, MM1S, OPM2 and INA-6 cells, 48h after G-1 treatment. Dot plots are representative of the percentage of apoptotic cells from an independent biological replicate (n = 3). Figure S3. Densitometric analysis of Sp1, MCL1, CDK6 and GPER1 protein fold change of expression in NCI-H929 cells treated for G-1 for 24 h. GAPDH was used as loading control.
