# Supplementary material for: GPER1 Activation Exerts Anti-Tumor Activity in Multiple Myeloma
Source: Cells. 2023 Sep 7;12(18):2226. doi: 10.3390/cells12182226 (PMC10526814; doi:10.3390/cells12182226)
Supplement: Supplementary file 1 [file cells-12-02226-s001.zip › cells-2578421-supplementary.pdf]

Article

GPER1 activation exerts anti-tumor activity in multiple myeloma

Maria Eugenia Gallo Cantafio[1], Roberta Torcasio [1,3], Francesca Scionti[2], Maria Mesuraca[1], Domenica Ronchetti[4], Mariaelena Pistoni[5], Dina Bellizzi[6], Giuseppe Passarino[6], Eugenio Morelli[7], Antonino Neri[8], Giuseppe Viglietto[1], and Nicola Amodio[1,*]

		IC_{50} ±SD (µM)
**MM cell lines**	AMO wt	0.84±0.05
	AMO-BZB	0.863±0.06
	AMO-CFZ	0.877±0.1
	MM1S	0.367±0.05
	MM1R	1.077±0.1
	INA6	0.726±0.07
	U266	0.744±0.08
	NCI-H929	0.782±0.08
**MM primary cells**	MM pt#1	>2
	MM pt#2	>2
	MM pt#3	>2

Supplementary Table S1. IC_{50} values of G-1 in MM cell lines and primary cells from newly diagnosed MM patients. Cells were treated with different concentrations of G-1 for 48h, and cell viability assayed using the CTG method. IC_{50} values were calculated using GraphPad Prism 8 software and reported as mean of three independent experiments ±SD.

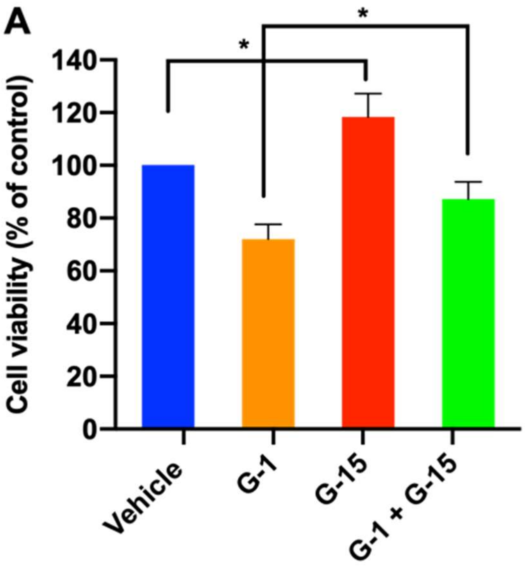

Supplementary Figure S1. Cell viability was assessed by CTG assay in NCI-H929 cells, 48h after 2 μM G-1 treatment, alone or in combination with 0.5 μM G-15. Histogram bars are representative of the percentage of viable cells compared to control. *$p < 0.05$.

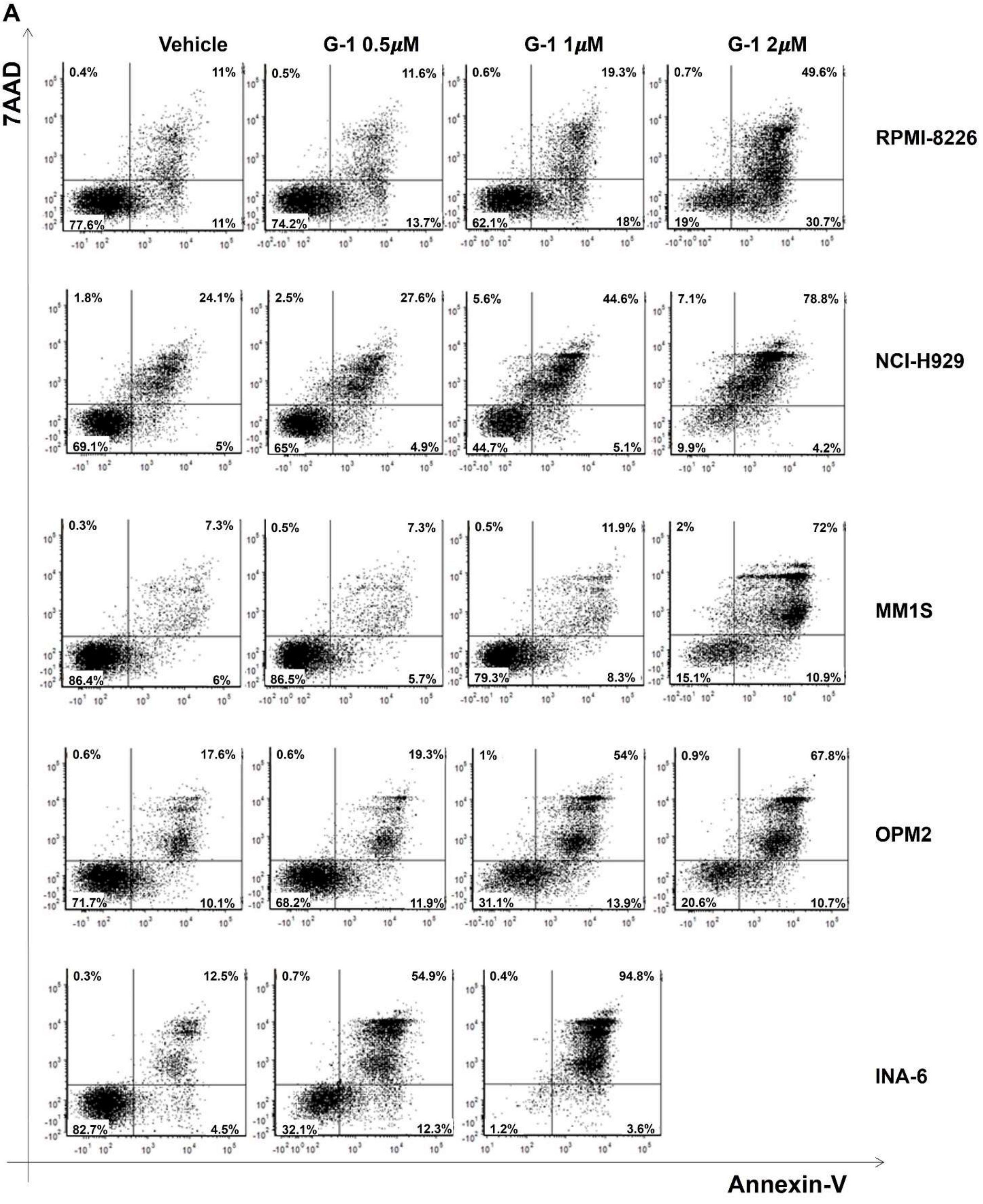

Supplementary Figure S2. FACS flow analysis of Annexin V/7-AAD stained RPMI-8226, NCI-H929, MM1S, OPM2 and INA-6 cells, 48h after G-1 treatment. Dot plots are representative of the percentage of apoptotic cells from an independent biological replicate (n = 3).

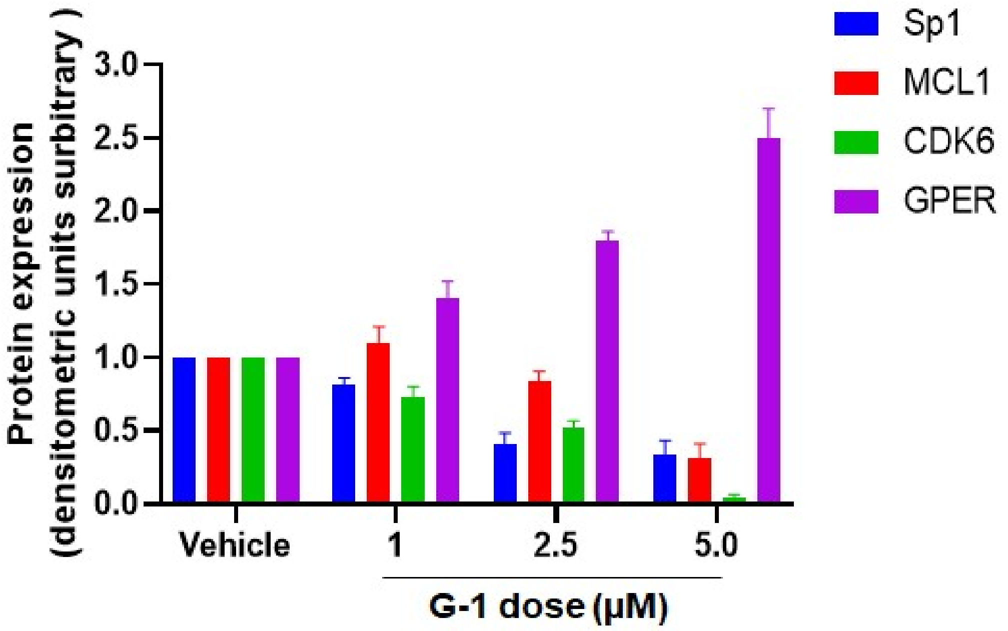

Supplementary Figure S3. Densitometric analysis of Sp1, MCL1, CDK6 and GPER1 protein fold change of expression in NCI-H929 cells treated for G-1 for 24h. GAPDH was used as loading control.
